# Global Implications From the Rise and Recession of Telehealth in Aotearoa New Zealand Mental Health Services During the COVID-19 Pandemic: Mixed Methods Study

**DOI:** 10.2196/50486

**Published:** 2023-09-22

**Authors:** Benjamin Werkmeister, Anne M Haase, Theresa Fleming, Tara N Officer

**Affiliations:** 1 School of Health Te Herenga Waka - Victoria University of Wellington Wellington New Zealand; 2 Department of Psychological Medicine University of Otago (Wellington) Wellington New Zealand; 3 Te Whatu Ora Wellington New Zealand; 4 School of Nursing, Midwifery, and Health Practice Te Herenga Waka - Victoria University of Wellington Wellington New Zealand

**Keywords:** telehealth, mental health services, Aotearoa New Zealand, mixed methods research, clinician, COVID-19

## Abstract

**Background:**

The COVID-19 pandemic accelerated the adoption of telehealth services for remote mental health care provision. Although studies indicate that telehealth can enhance the efficiency of service delivery and might be favored or even preferred by certain clients, its use varied after the pandemic. Once the pandemic-related restrictions eased, some regions curtailed their telehealth offerings, whereas others sustained them. Understanding the factors that influenced these decisions can offer valuable insights for evidence-based decision-making concerning the future of telehealth in mental health services.

**Objective:**

This study explored the factors associated with the uptake of and retreat from telehealth across a multiregional outpatient mental health service in Aotearoa New Zealand. We aimed to contribute to the understanding of the factors influencing clinicians’ use of telehealth services to inform policy and practice.

**Methods:**

Applying an interpretive description methodology, this sequential mixed methods study involved semistructured interviews with 33 mental health clinicians, followed by a time-series analysis of population-level quantitative data on clinician appointment activities before and throughout the COVID-19 pandemic. The interviews were thematically analyzed, and select themes were reframed for quantitative testing. The time-series analysis was conducted using administrative data to explore the extent to which these data supported the themes. In total, 4,117,035 observations were analyzed between September 2, 2019, and August 1, 2022. The findings were then synthesized through the rereview of qualitative themes.

**Results:**

The rise and recession of telehealth in the study regions were related to 3 overarching themes: clinician preparedness and role suitability, population determinants, and service capability. Participants spoke about the importance of familiarity and training but noted differences between specialist roles. Quantitative data further suggested differences based on the form of telehealth services offered (eg, audiovisual or telephone). In addition, differences were noted based on age, gender, and ethnicity; however, clinicians recognized that effective telehealth use enabled clinicians’ flexibility and client choice. In turn, clinicians spoke about system factors such as telehealth usability and digital exclusion that underpinned the daily functionality of telehealth.

**Conclusions:**

For telehealth services to thrive when they are not required by circumstances such as pandemic, investment is needed in telehealth training for clinicians, digital infrastructure, and resources for mental health teams. The strength of this study lies in its use of population-level data and consideration of a telehealth service operating across a range of teams. In turn, these findings reflect the voice of a variety of mental health clinicians, including teams operating from within specific cultural perspectives.

## Introduction

### Background

The COVID-19 pandemic and nationwide lockdowns have negatively affected mental health and well-being worldwide [1,2]. In Aotearoa New Zealand, difficulties in employment, socialization, financial, and housing stressors added to the psychological toll [3,4]. People adapted by making lifestyle changes, adjusting how they engaged with family and community support, and adopting new ways of accessing mental health care [5]. During the 2020 COVID-19 lockdowns in New Zealand, there was increased psychological distress [6] and increased overdose and self-harm presentations [7]. Such changes in mental distress mirror those found internationally, with research suggesting that these changes have led to long-term increases in mental health service demands [8]. Internationally, mental health services in many countries are now entering a postpandemic environment, with predicted increases in anxiety, posttraumatic stress disorder, and mood disorders, because of challenges with isolation, loneliness, job loss, and financial hardships [9-11].

Leading up to the COVID-19 pandemic, there has been a growing body of evidence for the effectiveness of telehealth in mental health settings [12-14]. Telehealth services have been used mainly for managing depression and posttraumatic stress disorder [15-17] and for improving service access to rural and underresourced services [18-20]. Telehealth service provision has been used successfully in geriatric, child, and adolescent populations [21,22]. Telehealth has the potential to improve service delivery [23], access, and social support while remaining cost-effective [24]. In New Zealand, client perspectives on the pandemic suggest that having a relationship with their clinician, the ability to create a safe space, and the option to choose methods of service delivery improved their experience with mental health service delivery via telehealth [25]. Perspectives from clinicians highlight the increasing workload and burnout, lack of telehealth training, inadequate infrastructure, and the need to support clinicians to use telehealth effectively [26-28].

Globally, to minimize the COVID-19 exposure, before widespread vaccination was available, mental health services adopted a variety of telehealth initiatives, including telephone, SMS text messaging, audioconferencing, and videoconferencing services [9,29]. Despite growing evidence for telehealth and its pandemic-related uptake, some services rapidly retreated from telehealth when circumstances no longer required it for routine practice [30], whereas others are working toward its sustainable implementation [31,32]. Given the growing body of literature on the potential effectiveness of telehealth, understanding why some mental health services rapidly retreated from a potentially acceptable, effective, and efficient model is timely to inform the future use of telehealth outside the pandemic and prevent the creation of new clinician-created service delivery barriers. We can learn from factors associated with these changes, such as (1) differences in uptake between populations [33] and (2) barriers to effective implementation [34-36].

### Aotearoa New Zealand Context

Specialist mental health and addiction services provide treatment to clients facing the greatest need for mental health support. Despite increases in ring-fenced funding in New Zealand [37], clients referred to specialist services experience difficulty in accessing these services in a timely manner, with national targets for timeliness and client numbers not being met [38] and Indigenous Māori populations facing worse health outcomes than non-Māori populations [39]. There has been a steady increase in clients accessing services [38] and poor clinician retention [40], creating concerns about clinician burnout and workforce support [41]. Furthermore, limited funding has imposed resource constraints [40], hindering service provision and development.

New Zealand’s initial pandemic response was one of the most stringent worldwide [6,42]. Between March 21, 2020, and December 2, 2021, the country operated a 4-stage *alert level* system; in the most stringent lockdown levels (level 3 and 4), travel was largely restricted to essential services such as groceries and health care [43]. Movement between alert levels was governed by the extent of viral spread, meaning that individual regions often had different alert levels. Later (December 2021 to mid-September 2022), the country used a 3-tier “traffic light” system, where the level of restriction was governed by infection rates and vaccination coverage [43]. Following this, New Zealand largely returned to its pre–COVID-19 practices. Alongside these changes, New Zealand began significant health system reform, with the aim of developing a more nationally integrated system to improve access and improved equity for Māori populations [44].

The aim of this study was to identify factors that contributed to the uptake of and retreat from telehealth services, using the perspectives of mental health service clinicians and synthesizing findings together with appointment activity data.

## Methods

### Mixed Methods Approach

**Figure 1:**
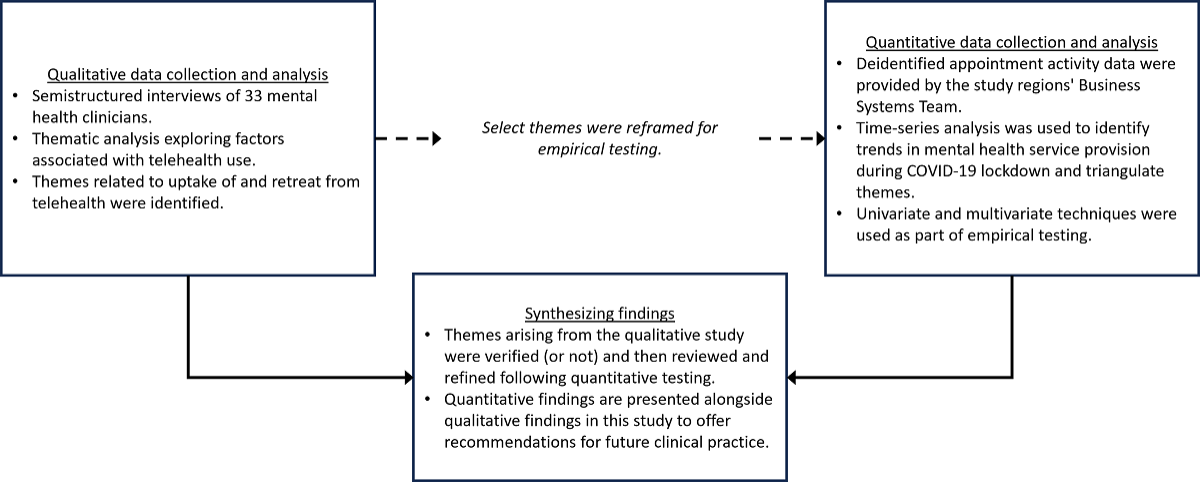
Flowchart of the study design.

This exploratory sequential mixed methods study used semistructured interviews with mental health clinicians, who provided telehealth care in outpatient mental health service settings, and analysis of quantitative appointment activity data during the pandemic (Figure 1). Findings from these interviews regarding COVID-19 clinician lockdown experiences [28] and client telehealth experiences [25] have been previously published.

 This study focused on the findings from interviews, exploring the factors contributing to the uptake of and retreat from telehealth. Select themes arising from interviews that lent well to quantitative testing were reframed for triangulation against quantitative data, facilitating the sequential synthesis and verification of themes, in line with the research of Shorten and Smith [45]. A time-series analysis was conducted to identify differences in audiovisual, telephone, and in-person service uptake for each tested theme.

### Ethical Considerations

Ethics approval was obtained from the Victoria University of Wellington Human Ethics Committee (*#*28808), and the study was endorsed by the relevant Research Advisory Group – Māori (#765). Written informed consent was obtained from all interview participants using a standardized information sheet and consent form. Participant interviews were deidentified before the analysis. Consent forms, interview transcripts, and demographic data were securely stored on the Te Herenga Waka—Victoria University of Wellington servers. Following the completion of interviews, a morning or afternoon tea was provided to all participating outpatient teams as a gratitude for the participant’s time; this was funded by the wider research grant budget. Quantitative appointment activity data were provided following ethics committee approval and were used in line with a Memorandum of Understanding between the research team and health service providers.

### Methodology

This research was guided by Thorne et al's [46] interpretive description methodology. Interpretive description was chosen, as it provides a foundation to investigate real-world situations by immersing the researcher in the phenomenon to produce logical conceptual descriptions. As a result, the researcher may reflect on the accumulated experience of research participants, and empirical evidence, to formulate a thematic or conceptual description that can be translated into practice [47]. Although traditionally used as a methodology for qualitative research, interpretive description is increasingly being used in the mixed methods space [48,49]. In this paper, quantitative findings have been used to add weight to the experiences described qualitatively by mental health clinicians.

### Study Location

This research was conducted in the publicly funded health system of 3 regions of New Zealand that work under 1 administrative umbrella. A total of 22 outpatient teams provided mental health services for these regions; each team comprised clinicians (including a mixture of physicians, nurses, clinical psychologists, social workers, occupational therapists, cultural workers, and team leaders). Team size ranged between 5 and 26 clinicians. The regions included subspecialty teams offering culturally specific services tailored to Indigenous Māori and Pacific populations. These included services aimed at offering traditional healing practices and Kaupapa Māori (Māori approach, led by Māori clinicians) and Pacific models of health delivery. These services aim to bring together culturally safe care within therapeutic environments.

### Semistructured Interviews

#### Overview

Full details of this method of data collection have been described in our earlier work [28]. Interviews focused on clinician experiences of health service delivery during the pandemic and mirrored parallel work into client experiences of service delivery [25]. Outpatient mental health clinicians who had delivered telehealth-based services at least once during the 2020 COVID-19–related lockdowns were invited by the team leaders of the 22 teams to participate. A total of 33 clinicians participated in semistructured one-to-one interviews lasting between 40 and 60 minutes between November 2020 and February 2021. Recruitment ceased when no new information emerged and when data offered sufficient variability [48].

Participant demographic details were collected using a standard questionnaire (Multimedia Appendix 1), and familiarity with telehealth was recorded on a 5-point Likert scale. The questionnaire used the same unique identifier as the participant transcripts and did not contain participant names.

#### Positioning and Reflexivity

The lead author conducted the thematic analysis and was placed as a research insider. BW is a psychiatric registrar (medical physician) working in the study region. Embracing an interpretive description methodology allows practitioner-researchers to be “seen,” as their professional knowledge drives aspects of data analysis [48]. Consequently, tensions between researcher and practitioner roles require balancing [47]; continuous critical reflection is necessary to prevent disciplinary biases from coming to the fore, as highlighted by Thorne [48]. Regular meetings with the wider research team were implemented to triangulate findings with the wider project and create an “external critic” [48], as the wider research team did not comprise mental health clinicians.

#### Analysis

Thematic analysis was chosen because it is suited to larger qualitative data sets, bringing together varied perspectives and the ability to organize findings in a clinical context [50]. Deidentified interview transcripts were uploaded to NVivo (version 12; QSR International) to support the analysis. Thematic analysis was conducted iteratively in line with Braun and Clarke [50], meaning that themes were refined as new instances of meaning arose through subsequent interviews, and later interviews evolved through this analysis process. Transcripts were reviewed and codes were developed based on individual instances of meaning. The codes were then grouped together based on shared meanings to develop themes. The emerging themes were then discussed within the wider research team for triangulation. After the final interview, themes were reviewed by the wider research team to check for consistency. Following quantitative analysis (see the *Activity Data* subsection), these themes were rereviewed while considering quantitative findings and as part of the mixed methods synthesis process. These rereviewed themes are presented in the *Results* section.

### Reframing Themes for Quantitative Testing

Quantitative testing can be used to triangulate and support qualitative themes as described by Dolan et al [49] and Mays and Pope [51]. A total of 6 preliminary subthemes arose during the thematic analysis that related to a clinician’s decision to use telehealth (ie, supporting the discussion of the uptake of and retreat from telehealth use). Within these 6 themes, 4 aspects were well suited for quantitative testing. These were reframed before testing using the quantitative data, as presented in Textbox 1.

**Reframed themes for testing.**

1. Clinicians were more familiar with service provision by telephone than audiovisual (AV) mode.
2. There was differential uptake of AV and telephone services by the clinician role.
3. Demographic factors influenced AV and telephone service use.
4. The convenience of telehealth services improved access to mental health services.

### Activity Data

#### Overview

Deidentified data (in which clinician and client names were replaced with a unique identifier) were obtained through the Business Systems Team in the study regions. As the only available digital record of outpatient mental health clinician activities across the study regions, activity data from the 3 regions were used to test the reframed themes. This data set represented population-level data and included all clinical activities (4,117,035 observations) undertaken by outpatient mental health clinicians in the study regions between September 2019 and August 2022 to allow comparison of telehealth use between prelockdown, lockdown, and postlockdown periods. Key data fields include the following:

- Mode of service delivery (eg, in person, audiovisual including videoconferencing, or telephone)
- Client demographic data
- Type of activity undertaken
- Duration of activity (separated into direct, indirect, and travel time)
- Date or time of the activity undertaken

#### Analysis

The data were analyzed using R/RStudio (×64 version 4.1.2; Posit Software). Univariate techniques (eg, measures of variability and central tendency) were used to check for internal consistency and identify outliers or gaps in the data. Outliers >3 SDs from the mean and incomplete entries were documented and sent to the Business System Team for verification and correction. The data set was cleaned using R/RStudio at 4 major stages (Textbox 2).

**Data cleaning and assumptions.**

**Remove unnecessary entries**

- Duplicate rows and redacted information were deleted.
- Fields and categories were not needed for analysis.
- Teams, clinicians, and activities were not undertaken by mental health clinicians.

**Standardize fields**

- Each tranche of data had new categories for ethnicity and gender. These were standardized back to the grouping provided in the original data tranche. Ethnicity data was expanded in 2021 to include “Asian,” “Pasifika,” and “Other”. This was standardized by grouping “Asian,” “Pasifika,” and “Other” as “Non-Māori,” meaning that data showed only 2 categories “Māori” and “Non-Māori.” Gender data were expanded to include “Gender Diverse” in 2021. This was standardized by combining “Gender Diverse” and “Unknown” categories into a “Non-binary” category.

**Group categories and activities**

- The original data set included too many categories to provide meaningful analyses. As a result, outpatient teams were grouped by specialty (Māori, Pasifika, Adult, Child and Adolescent, Addictions, Crisis Resolution [CRS], Forensic, Psychogeriatric, and Subspecialty). Teams operating within a subspecialty provided similar services to teams sharing their subspecialty.
- Activities were grouped by activity type (client contact, family contact, client and family contact, group activity, notes, and liaison with another professional).
- Delivery modes were grouped into telephone, audiovisual (AV), in person, did not attend (DNA), and cancellation.
- Clinicians only provided services by AV, telephone, or in person. There were no categories for hybrid approaches (clinicians present in person and AV). Email and text communications were not recorded in the data set.

**Assumptions**

- Outpatient clinicians cannot undertake multiple activities simultaneously. To manage this issue, concurrent activities sharing a unique clinician ID were weighted and group activities were coded separately for each client by a single clinician ID. Concurrent activities sharing a unique client ID, but multiple clinician IDs were not weighted.
- Activities with 0-min total duration (direct min + indirect min + travel min) were recorded as invalid and removed. This was assumed to be a coding error as an activity with no duration does not occur. Exception: DNA and cancellations were not removed as they are a record of events that did not occur.
- When direct minutes were=0, the value for direct minutes was replaced by the value for indirect minutes.
- Direct minutes record the duration of activity undertaken, and indirect minutes record duration of work associated with the activity undertaken.
- When both direct minutes and indirect minutes were 0, then the activity was assumed to be transport only.

**Define lockdown periods and workdays**

- Periods were created based on the New Zealand Government’s Official COVID-19 alert levels for the study region (New Zealand Government 2020).
- Alert levels 1 and 2 were classified as period 1, as service restrictions placed on clinicians during these levels were similar.
- In December 2021, the alert level system was discontinued and replaced with a traffic light system. The period from December 2021 to August 2022 was classified as period 1 as the traffic light system had equivalent restrictions to alert levels 1 and 2.
- Alert levels 3 and 4 were not combined as restrictions placed on clinicians differed and reflected an evolving response to lockdown.

**Assumptions**

- Outpatient clinicians are employed to work between the hours of 8 AM and 5 PM. Exception: physicians and CRS clinicians have additional shifts to cover the remaining hours. Data were filtered to include any activity starting between 8 AM to 5 PM.
- Outpatient clinicians do not work on weekends or public holidays. Exception: physicians and CRS clinicians also work weekends and public holidays. Data were filtered to exclude weekends and public holidays observed in the study regions.
- Regular workdays were defined as 8 h in length, and outpatient clinics were programmed to close by 5 PM. Clinicians could work additional duties (not regular outpatient duties), the longest duration of safe working hours was assumed to be 1 additional shift (total 16 h/d). Individual activities longer than 8 h were scaled back to 8 h. Activities longer than 16 h were considered outliers or erroneous data collection and removed from the analysis.

The initial analysis involved gaining familiarity with the data set. This was achieved by generating time-series graphs of the number of clients receiving treatment and clinicians providing care, activities undertaken, and average activity duration. These were used to understand overarching service provision and clinician workload trends. Missed appointments were reviewed to identify service inefficiencies. The weekly means of the daily average cancellations and nonattendance (did not attend) were calculated and plotted on time-series graphs for each COVID-19–related lockdown period (Table 1).

The second stage of the analysis quantified trends in audiovisual, telephone, and in-person service delivery modes. The dependent variables chosen for the analysis were measures of clinical activity frequency (client consultation, family consultation, and group activity) and duration. Trends in delivery mode were visualized by calculating weekly running averages for each dependent variable and plotting them on a time-series graph. Summary tables were created for the changes in the dependent variables for each delivery mode across each COVID-19 lockdown period.

In the semistructured interviews, participants identified that client demographics (age, ethnicity, and gender); clinician factors (role and team subspeciality); and the COVID-19 period (effect of lockdown) affected the uptake of telehealth services. The third stage of the analysis quantified the effects of these independent variables on the trends identified in the second stage of the analysis. The independent variables were selected to test the preliminary themes listed in Textbox 1.

To quantify the effect of each independent variable on the service delivery mode, the independent variables were initially analyzed separately. For each independent variable, the mean daily average activity count was calculated and filtered by the delivery mode and COVID-19 period. Descriptive statistics were used to compare the changes in activity counts related to each independent variable.

**Table 1:** COVID-19–related lockdown periods.

COVID-19–related restriction level	Start date (time)	End date (time)	Lockdown status
0^a^	September 2, 2019 (12 AM)	March 21, 2020 (12 AM)	Before lockdown
1^b^ (1)^c^	March 21, 2020 (12 AM)	March 23, 2020 (1:30 PM)	Alert levels started
3 (1)	March 23, 2020 (1:30 PM)	March 25, 2020 (11:59 PM)	First lockdown
4 (1)	March 25, 2020 (11:59 PM)	April 27, 2020 (11:59 PM)	First lockdown
3 (2)	April 27, 2020 (11:59 PM)	May 13, 2020 (11:59 PM)	First lockdown
1 (2)	May 13, 2020 (11:59 PM)	August 17, 2021 (11:59 PM)	Out of lockdown
4 (2)	August 17, 2021 (11:59 PM)	August 31, 2021 (11:59 PM)	Second lockdown
3 (3)	August 31, 2021 (11:59 PM)	September 7, 2021 (11:59 PM)	Second lockdown
1 (3)	September 7, 2021 (11:59 PM)	August 1, 2022 (11:59 PM)	No further lockdowns

^a^Period 0 represents time before lockdown.

^b^COVID-19 alert levels 1 and 2 were combined into period 1 because of similar loosening restrictions for clinicians. Levels 3 and 4 were not combined, as clinician restrictions differed, which reflected an evolving lockdown response. On December 2, 2021, the alert level system was discontinued and replaced with the traffic light system, which was discontinued on September 12, 2022 [43].

^c^Subsequent occurrences of a period are labeled (n) in chronological order.

## Results

### Demographics of Interview Participants

Interview participants (N=33; female participants: n=23, 70%; male participants: n=10, 30%) constituted a range of professional disciplines and had been working in mental health services for 2 to 40 years. The participants were predominately New Zealand European (22/33, 67%), with 5 (15%) from Māori population, 2 (6%) from Pacific people, and 4 (12%) from other ethnicities. Participants were generally evenly distributed between the ages of 25 and >65 years, with a modal age range of 45 to <55 years (10/33, 30%; Table 2).

**Table 2:** Participant clinical role (N=33).

Role	Participant, n (%)
Physician	7 (21)
Nurse	6 (18)
Clinical psychologist	5 (15)
Team leader	5 (15)
Social worker	4 (12)
Occupational therapist	2 (6)
Cultural therapist and Kaumātua^a^	2 (6)
Psychotherapist	1 (3)
Case manager	1 (3)

^a^Kaumātua are respected Māori elders.

### Overview

A total of 6 subthemes arose from the qualitative analysis that influenced clinician decisions to use telehealth services in the study regions. These were grouped under 3 overarching themes, focusing on clinician roles, population characteristics, and system readiness as shown in Table 3. Themes were presented alongside quantitative data to strengthen the study findings, in line with Thorne [48].

**Table 3:** Themes, subthemes, and the related to reframed tested themes seen in Textbox 1.

Overarching themes and subthemes			Related reframed themes
**Clinician preparedness and role suitability**			
	Familiarity and training	1	
	Specialist role considerations	2	
**Population determinants**			
	Age, gender, and ethnicity	3	
	Clinician flexibility and client choice	4	
**Service capability**			
	Access to services and digital exclusion	N/A^a^	
	Usability	N/A	

^a^N/A: not applicable.

### Clinician Preparedness and Role Suitability

#### Overview

Clinicians had varied prior experience with audiovisual and telephone services and differential access to training. Furthermore, there were activities, unique to clinical roles, that did not lend well to audiovisual or telephone service provision, limiting telehealth uptake by these clinicians. Together, these factors influenced clinician feelings of preparedness and the perceived appropriateness for delivering mental health services via telehealth.

#### Familiarity and Training

Participants in the qualitative study had wide-ranging experience with telehealth service provision. All participants indicated familiarity with and regular use of telephone service provision. However, the participants were less familiar with using audiovisual technology (to the statement “I consider myself very familiar with videoconferencing,” the 33 participants responded as strongly disagree: n=0; disagree: n=2, 6%; neutral: n=7, 21%; agree: n=18, 55%; strongly agree: n=6, 18%). Participants clarified that they were familiar with using audiovisual technology outside their clinical role for social contacts but lacked experience and training in using audiovisual technology to provide therapeutic interventions and consultations. In interviews, only 3 participants described their experiences with audiovisual telehealth service provision before the first New Zealand COVID-19 lockdown.

Most clinicians described a rush to familiarize themselves with the technology, either during or immediately preceding the lockdown. There were few individuals who had experience with telehealth from previous employment; of them, all indicated that their experience improved their readiness to use audiovisual services:

I was shocked by how little anybody knew about it. We talked about telehealth for years...I’d worked in Australia for many years, telehealth was routine practice. When I arrived [in New Zealand], I was surprised that we weren’t using it. But there wasn’t the energy, interest, confidence, to use it.

Participant 4

Participants experienced difficulty in accessing formal training on the use of audiovisual services because of a lack of training programs available through their employer. Most clinicians described poor access to training resources and procedures for establishing audiovisual services. Some clinicians who took an interest in audiovisual service provision engaged with web-based education sessions and reviewed the available research to develop the skills needed to engage using audiovisual technology. Furthermore, as the lockdown progressed, many participants felt overwhelmed by a flood of information on audiovisual service use, encountering conflicting information on what telehealth could be used for and how to use the service effectively. They felt that this tainted their experience with audiovisual services and contributed to abandoning audiovisual service use when they were out of lockdown:

Nobody sent any information about how to use technology. No one. I’m part of lots of organisations, especially with nursing, and I didn’t receive anything in regards to how we utilise our tools and how to work better at home and stuff like that. That stuff started coming out more closer to when we were coming out of lockdown.

Participant 9

Physicians and clinical psychologists were able to access training on how to conduct audiovisual appointments through their professional organizations (The Medical Council of New Zealand, Medical Protection Society, Royal Australian and New Zealand College of Psychiatrists, and New Zealand College of Clinical Psychologists). Participants who had received formal training felt more comfortable using audiovisual services and recognized that this influenced their ongoing use of audiovisual services:

I did several MPS [Medical Protection Society] evening webinars on telehealth...You can’t just get the technology in without the training as well and the support.

Participant 14

**Figure 2:**
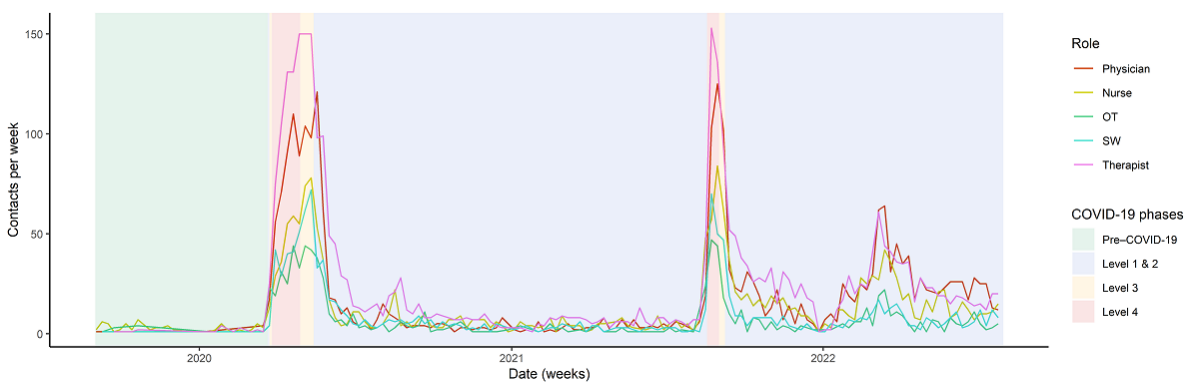
Audiovisual uptake by role. OT: occupational therapist; SW: social worker.

This is supported by quantitative findings, as physicians and clinical psychologists (therapists) conducted more audiovisual consultations during the study period than other specialists, who had limited access to training, as shown in Figure 2.

#### Specialist Role Considerations

Physicians and clinical psychologists conducted psychological therapy and complex assessments, which were unique to their clinical roles. The common element required for these services was the need to observe the client and conduct a mental state examination. Clinicians felt that in the absence of in-person service delivery during the lockdown, audiovisual services were well suited for these clinical activities. However, similar to other clinician groups, they felt that in-person service provision was preferred, resulting in limited ongoing telehealth use after the lockdowns:

The one I remember the most, which was a Zoom, seeing a young man at the police station, and really being able through the Zoom to actually bring affect into the interview...in the Zoom conversation with him, for the Mental Health Act...he sort of crumpled like a little boy and started getting tearful...I don’t think it was unsafe, it was amazing that you could do that on Zoom.

Participant 12

Participants identified that some activities that were specific to social workers, occupational therapists, and cultural workers did not translate well into audiovisual or telephone service delivery. This included home assessments, meaningful group activities, and accommodation and employment supports. These clinician groups had limited audiovisual uptake during the study period, as shown in Figure 2. As one clinician explained:

One of the things I love working in [Outpatient team] is all the creative stuff, we get to get outside and do wacky things, and essentially all of that was taken away.

Participant 25

**Figure 3:**
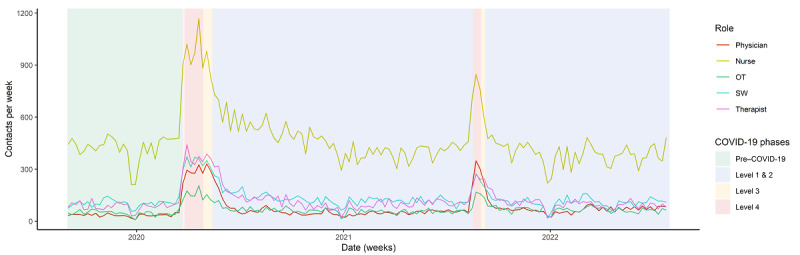
Telephone uptake by role. OT: occupational therapist; SW: social worker.

Nurses used telephone contact considerably more often than persons of other clinical roles (Figure 3). Participants described that the nursing role involved a large volume of short clinical contacts that lent well to telephone contact. The participants identified that telephone contact was useful for monitoring client progress through supportive contacts. However, they suggested that telephone contact was not adequate for in-depth assessments or for providing evidence-based therapies (such as cognitive behavioral therapy):

More for shorter interactions, not for the longer. If someone rang in unsettled, we’d use it for that. For planning face-to-faces, checking on briefer things but not for whole sessions. People required quite a lot of extra support because of the situation. A lot more talk therapy was delivered down the phone.

Participant 15

Clinicians described supporting their clients to attend appointments with their general practitioner to address physical illnesses for vulnerable clients. During the lockdown, clinicians reported that clients were unable to see general practitioners in person, which was particularly problematic for clients with severe eating disorders, who needed strict regular monitoring (eg, weight measurements, physical examination, blood tests, and electrocardiogram). Clinicians felt obligated to manage this responsibility in person, as this client group required close monitoring to avoid potentially life-threatening medical complications:

GPs were trying to keep people away, so it was a weird situation. We started a new clinic, which was just one of the nurses and she was basically just seeing people one-to-one and she had all the PPE stuff and she’d just get them in and weigh them and just ask them a really brief kind of like’ have you been binging, purging, using laxatives?

Participant 22

### Population Determinants

#### Overview

Clinicians identified specific determinants as likely contributing to the differential uptake of telehealth among clients and clinicians. For clients, it was hypothesized that differences in age, gender, and ethnicity may impact service use. In addition, the option of audiovisual and telephone services was considered as a way to improve service flexibility and attendance. In turn, clinicians emphasize the importance of providing a choice to clients.

#### Age, Gender, and Ethnicity

Participants identified a generational divergence between younger and older clinicians. They opined that younger clients and clinicians were more likely to have been exposed to audiovisual technology from a younger age and developed skills as they grew up, increasing their willingness to use audiovisual services in a health care setting. Furthermore, participants believed that older clients and clinicians were more likely to be unfamiliar with audiovisual technology and would find it more difficult to learn how to participate in new audiovisual services:

There will always be a generation gap problem. Older clinicians struggling with all that and younger ones just doing it no problem. It’s a bit like typing, when you see people typing their notes now, how fast or slow they type on the keyboard.

Participant 16

**Figure 4:**
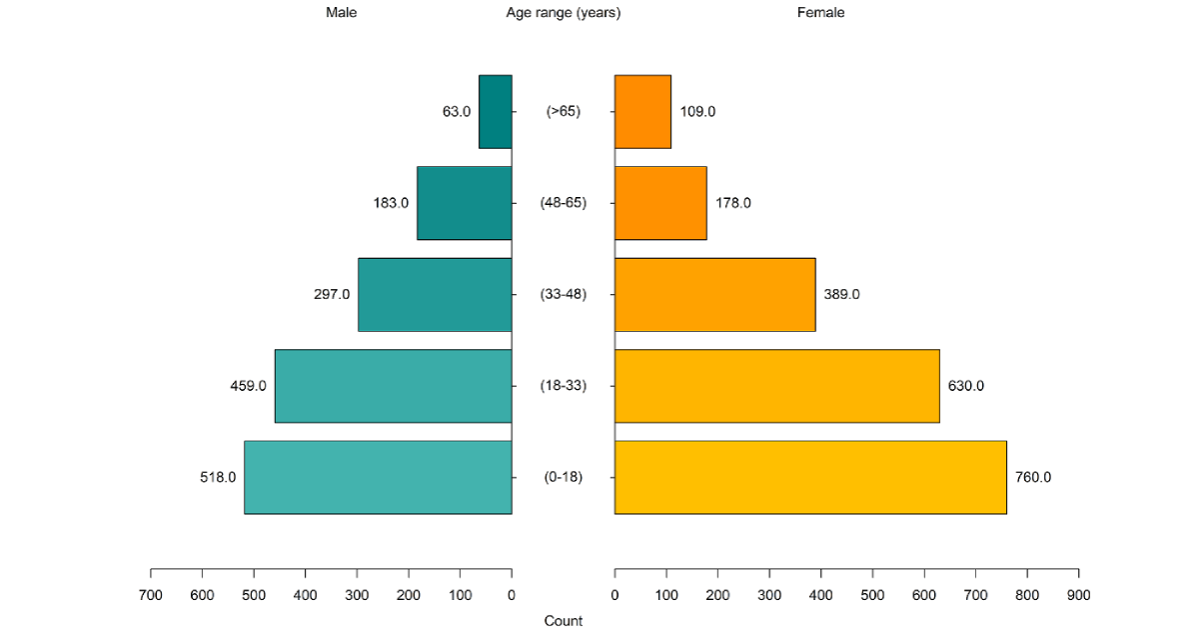
Age and gender distribution of audiovisual service provision.

**Figure 5:**
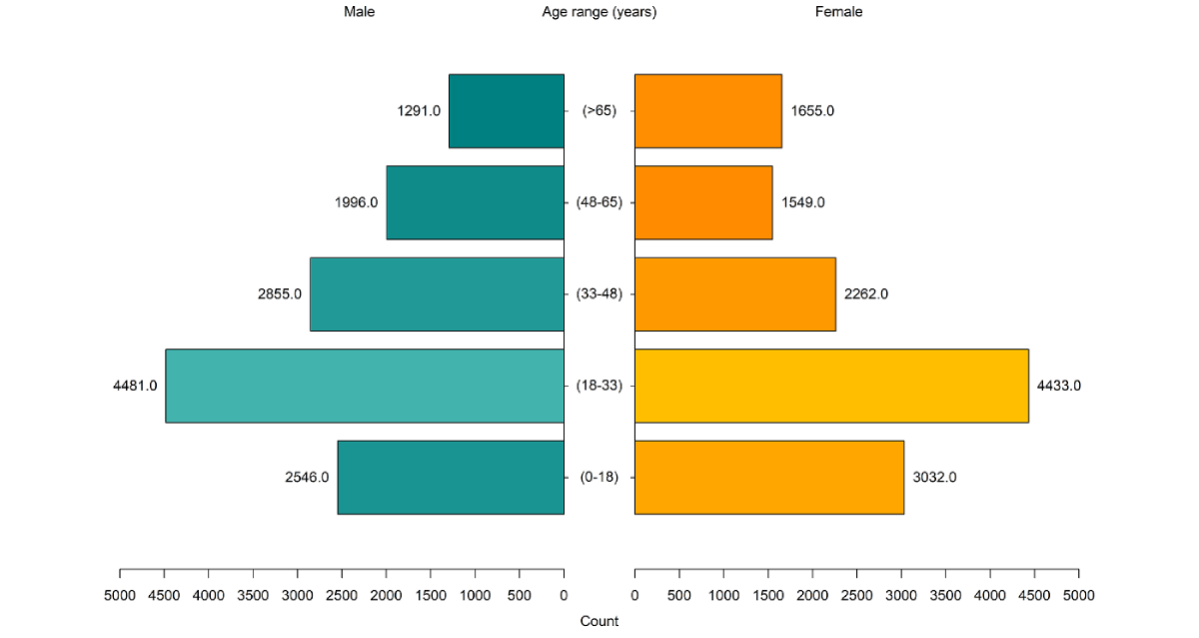
Age and gender distribution of in-person service provision.

**Figure 6:**
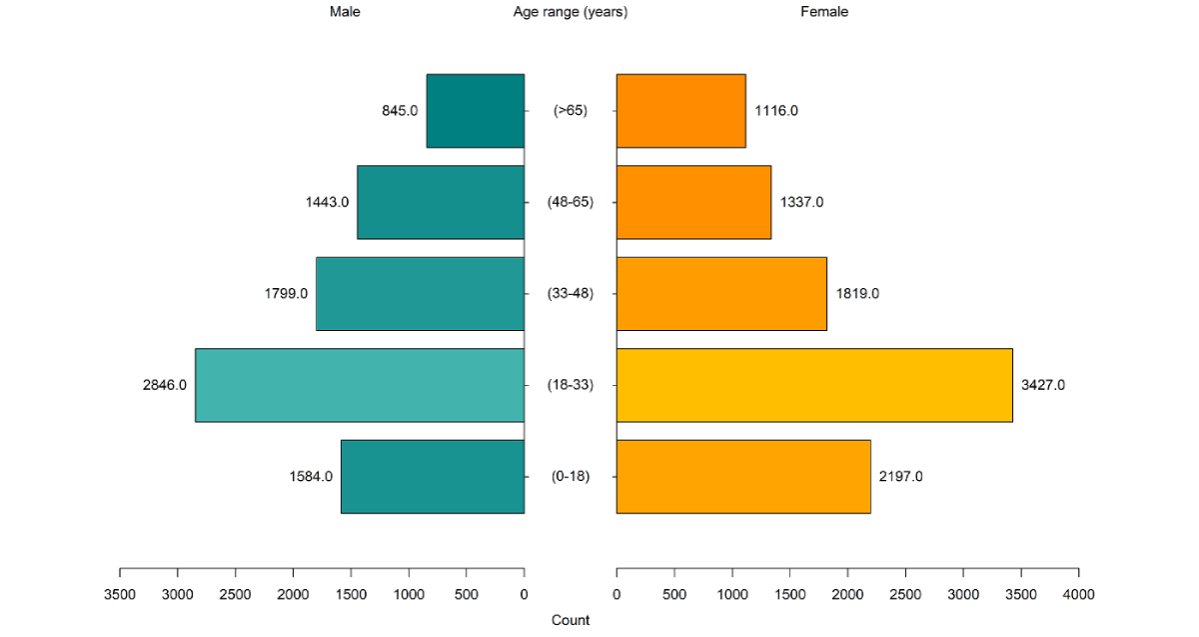
Age and gender distribution of telephone service provision.

This generational divergence is supported by quantitative findings. Comparing age and gender (male and female) distribution of audiovisual service provision (Figure 4) with those of in-person (Figure 5) and telephone (Figure 6) service provisions, there was an increased proportion of audiovisual use by clients aged between 0 and 18 years and a decreased proportion of audiovisual use in the age range of >65 years.

The in-person and telephone service provisions had similar age stratification. Most contacts were with clients in the age range of 18 to 33 years, with less client contact in the age range of 0 to 18 years.

The participants believed that gender did not influence telehealth uptake. The data set had 49.47% (13,195/26,673) of female participants, 50.14% (13,373/26,673) of male participants, and 0.39% (105/26,673) of nonbinary participants. However, there were slight differences in contact between the service modalities. There were more in-person contacts with male clients, with the exception of those aged >65 years, which reflects the reduced number of male clients in this age group. There were more telephone and audiovisual contacts with female clients, with the exception of those aged from 48 to 65 years, which contradicts clinician experiences.

Participants believed that Māori clients and clinicians would be less likely to use audiovisual services. Māori populations have unique health needs, beliefs, and practices [39,52,53]. Specialist Māori mental health services in the study regions used culturally appropriate models of care to holistically support the mental well-being of Māori clients. *Kaimahi* (Māori clinicians) identified client distrust of Western medicine because of issues such as colonization, racism, and social injustice. Furthermore, *kaimahi* recognized that the implementation of telehealth services could be challenging for Māori clients, because it could be perceived as forcing Māori clients to adopt a Western model of service provision:

I think there would be cultural barriers as well. Cultures who are already have legitimate concerns around interacting with the Western biomedical model. They’re still going to have those same concerns using telehealth.

Participant 4

Kaimahi felt that traditional Māori health practices and culturally important services did not translate well into a telehealth platform. Activities such as Mau rākau (traditional martial arts) and blessings by kaumātua are traditionally undertaken in person, and kaimahi felt that they did not have the same impact without a physical presence:

It’s activities like using Mau Rākau and a number of other cultural activities where we actually require a physical presence. It may be a little bit different in terms of the therapy that may be delivered by psychology and other forms of therapy. But from a Māori world view, the face-to-face has the value of being able to be more productive.

Participant 11

Approximately 25.99% (6932/26,673) of the clients in our data set were Māori clients. During periods of lockdown, Māori populations used a higher proportion of in-person services and a lower proportion of audiovisual and telephone services than non-Māori populations (Table 4). This aligns with participants’ experiences of Māori clients preferring in-person service provision and themes identified by the *kaimahi* participating in the study.

**Table 4:** Trends in service delivery by ethnicity.

Participants and service delivery mode		Period^a^								
		0	1 (1)^b^	3 (1)	4 (1)	3 (2)	1 (2)	4 (2)	3 (3)	1 (3)
**Māori participants, mean h/d (% of daily contacts)**										
	IP^c^	135.7 (89.9)	36.2 (82.9)	65.1 (65.6)	54.6 (48.8)	68.8 (52.1)	139.1 (86.5)	58.6 (53.3)	71.4 (56.8)	110.5 (84.8)
	AV^d^	0.9 (0.6)	0.2 (0.6)	1.5 (1.5)	11.4 (10.2)	15.4 (11.7)	3.6 (2.2)	15.9 (14.5)	19.8 (15.7)	4.6 (3.5)
	Telephone	14.4 (9.6)	7.2 (16.5)	32.7 (32.9)	45.9 (41.0)	47.9 (36.3)	18.2 (11.3)	35.5 (32.3)	34.6 (27.5)	15.2 (11.7)
**Other participants, mean h/d (% of daily contacts)**										
	IP	386.1 (90.1)	96.1 (73.0)	153.3 (47.9)	108.2 (34.7)	150.2 (37.0)	376.5 (86.2)	125.1 (38.2)	147.6 (41.2)	293.4 (82.5)
	AV	0.9 (0.2)	1.4 (1.1)	18.2 (5.7)	42.2 (13.6)	67.3 (16.6)	7.5 (1.7)	70.9 (21.7)	92.1 (25.7)	16.6 (4.7)
	Telephone	41.6 (9.7)	34.1 (25.9)	148.8 (46.5)	161.0 (51.7)	188.2 (46.4)	52.5 (12.0)	131.4 (40.1)	118.5 (33.1)	45.6 (12.8)

^a^COVID-19 periods are presented in chronological order as outlined in Table 1.

^b^Subsequent occurrences of a period are labeled (n) in chronological order.

^c^IP: in person.

^d^AV: audiovisual.

#### Clinician Flexibility and Client Choice

Participants suggested that fewer clients missed appointments (cancellations and nonattendance) during the COVID-19 lockdown. Participants speculated that during the lockdown-related social isolation, the need for additional support, reduced conflicting commitments, and convenience improved attendance.

**Figure 7:**
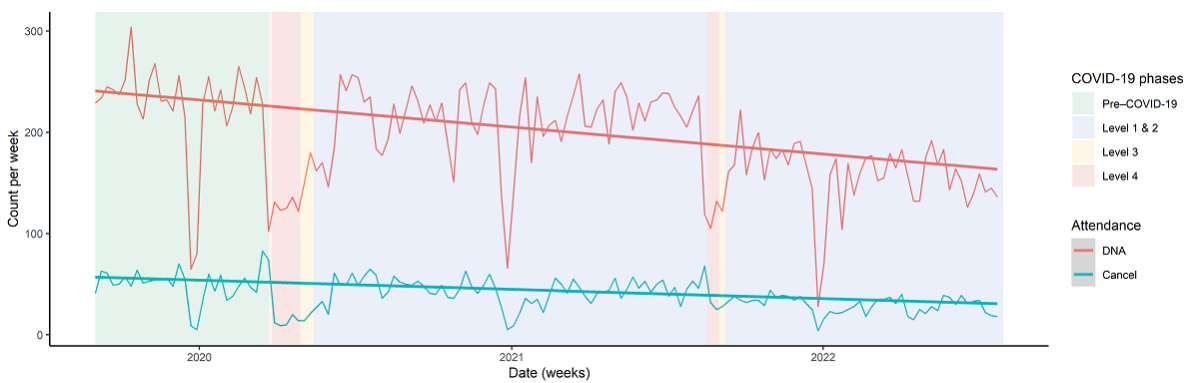
Incidence of nonattendance. DNA: did not attend.

Quantitative data supported this assertion (Figure 7) as seen by a reduction in the rate of missed appointments during the lockdown. This improvement was not sustained following the lockdown periods. However, there was a gradual downward trend in missed appointments following the initial lockdown in 2020, which could support participant claims of improved attendance because of convenience, improved familiarity, and some acceptance of telehealth service provision.

Participants identified a strong element of client and clinician choices when reflecting on telehealth service uptake. Clinicians and clients felt that during the lockdown, there was frustration around the limited options for service delivery. Participants voiced a preference for telephone contact over audiovisual telehealth service provision, and this was reflected in the higher uptake of telephone use than of audiovisual services. However, participants found that giving clients the choice of in-person, telephone, and audiovisual options improved their engagement. Furthermore, clinicians found that improving appointment flexibility by offering audiovisual or telephone appointments for convenience or when clients were unable to attend the clinic also improved client attendance:

I gave clients the choice, so they all chose. I do prefer Zoom, just being able to have some semblance of face-to-face contact and being able to read their faces and being able to do the non-verbal feedback that we would do...If people were on the phone always, I would encourage them to try and do a Zoom every now and then.

Participant 21

### Service Capability

#### Overview

COVID-19 brought about major changes to how services were delivered. These changes occurred without the mental health system necessarily being able to respond proactively. Participants emphasized the role telehealth played in changing access to services and concerns around digital exclusion. They also highlighted the difficulties experienced by clinicians in using telehealth for mental health service delivery, including conducting observations and managing therapeutic relationships.

#### Access to Services and Digital Exclusion

Some participants identified that their team had limited access to a psychiatrist, which limited their ability to respond to clients in crisis or difficult-to-reach clients. Some teams used audiovisual services in conjunction with a clinician attending in person to improve access to a psychiatrist. One participant described the following:

I got Zoomed to do a mental health review which occurred somewhere on a doorstep down an alleyway...I think having access to someone to give advice at short-notice is really good.

Participant 14

This participant continued to explain that access to a psychiatrist for review was often necessary in crisis assessments and mandatory if compulsory assessment and treatment under the Mental Health Act was considered. This hybrid model of care improved access to a psychiatrist, while another clinician was able to physically contain client risk.

Participants proposed that audiovisual service provision could be used to reduce wait times for service access and to improve access to initial assessments for clients referred to specialist services. The mean wait time (from referral to initial assessment by a clinician) before lockdown was 74.1 (SD 113.0) days, decreasing to 66.9 (SD 87.7) days during COVID-19 lockdown and decreasing to 15.7 (SD 12.9) days during alert levels 1 and 2. Participants described delaying initial appointments for referrals received during the lockdown until the return to alert levels 1 and 2. This could help explain the marked decrease in mean wait time during levels 1 and 2, as clinicians began seeing new clients referred during lockdown. Furthermore, there was a reduction in the mean daily referral rate from 8.77 (SD 8.93) referrals before the lockdown to 7.02 (SD 7.02) referrals during the lockdown and 8.66 (SD 8.89) during alert levels 1 and 2. This could help explain the slight decrease in wait time during the lockdown as clinicians caught up with lower-priority referrals:

I think so, because you know how people will be waiting for ages for CRS [Crisis Resolution Service]. Sometimes I think if we could actually use telehealth more, we’d be able to respond faster...say if you presented to your GP today, if I'm able to say, can I Zoom in with [clinician] and just triage it a bit more.

Participant 23

Participants proposed that audiovisual services could improve access to specialist mental health services by removing geographic barriers to accessing treatments and travel costs and facilitate access to services from the client’s home. One participant explained as follows:

I think for a sub-population of our patients, to save money and become more efficient, and easily accessible from home for them as well, that is an advantage.

Participant 19

The mean hours that clinicians spent traveling reduced from 56.7 (SD 15.7) hours before lockdown to 24.1 (SD 8.2) hours during lockdown and 47.9 (SD 13.8) hours during alert levels 1 and 2, owing to the reduction in in-person activities. The available data set did not record client transport for appointments or transport costs to clients or mental health services.

Participants described a variety of barriers to accessing telehealth services. Participants found that they lacked the basic resources to undertake audiovisual appointments during lockdowns. Clinicians required computer devices that could run audiovisual software with adequate resolution and sound quality, headsets to improve privacy, video cameras of adequate quality, and a private space to undertake assessments. Teams were not provided additional funding to purchase the necessary equipment, forcing them to make difficult choices within their budget, which limited the quality and availability of audiovisual equipment. Furthermore, the Māori and Pacific teams, who began the pandemic with hardware access issues, described disparities in funding for their teams, which they felt increased service inequities. Clinicians were forced to buy their own equipment, which some strived to afford only with considerable difficulty:

The team leader needed to buy some new technology, some better microphone and a better set up. It was like there’s hardly any money available to you to do this and by the time the gear arrived we were out of lockdown.

Participant 4

#### Usability

Participants described the need to address key clinical issues to enable effective audiovisual-based service provision. These included challenges in interpreting subtle visual cues, limited ability to conduct a mental state examination, difficulty in assessing risk (of suicide or violence), inability to monitor physical observations (such as blood pressure), and difficulty in forming therapeutic relationships.

Clinician observations are recorded as part of a mental state examination to provide objectivity in consultations and to quantify affect, psychotic phenomena, functional impairments, and neurological abnormalities. Participants lacked confidence in using audiovisual services for in-depth assessments, because it was difficult to interpret subtle visual cues, such as body language, facial expressions, motor movements, posture, gestures, and eye contact due to poor video quality. Clinicians explained that this reduced diagnostic confidence when using audiovisual services and resulted in less audiovisual service use. Clinicians were also less confident in assessing intoxication and whether clients were maintaining self-care (such as showering or toileting) via audiovisual mode, which drove them to rely on potentially unreliable client disclosure and collateral information:

If there was some kind of nuances in her facial expression or if I was, for example, wondering if she had something like tardive dyskinesia or something, it wouldn’t have been so clear because the picture wasn’t as clear as it would be to be in the room with her. There are certain things that would be harder to examine.

Participant 17

Participants faced technical difficulties with audiovisual technology, including compatibility issues with hospital software, poor audio and visual quality, and poor internet connectivity. This caused frustration among clinicians, as participants described stilted consultations, difficulty seeing or hearing their clients, and unnecessary interruptions during appointments. Clinicians felt that these technical issues made it more difficult to practice via audiovisual mode and influenced their decisions to return to in-person service provision:

They kept trying to do the audio through Citrix [clinical application] and it just didn’t work very well...You can’t get the audio through Citrix. And I thought, why didn’t somebody tell me that. I finally gave up, but I tried it and it was really frustrating.

Participant 12

Participants proposed that telehealth services were not suitable for clients with disorganized thought form, impulsivity, or a lack of capacity. Participants recognized that these capacity factors could hinder a client’s ability to understand and engage in appointments using telehealth services and make it difficult to assess client risk of suicide or violence toward others. Clinicians chose to see these clients in person during the lockdown to support decision-making and reduce barriers to understanding and engagement:

You need to be able to use a complex system like a computer and still have enough cognition to understand the concept of telehealth and that you talk to a virtual person. But a psychotic person, for example, is disturbed in thoughts and disorganised, you will very likely not be able to engage by themselves.

Participant 19

Clinicians continued to see clients in person during the lockdown for physical monitoring. Participants identified that clinicians working in general practice were lending equipment to their clients to allow them to self-monitor vital signs, minimizing the need for in-person contact during lockdown. Participants lacked the necessary equipment to set up similar mental health services. Clinicians required portable blood pressure monitors, temperature monitors, and pulse oximeters to lend to clients and provide instructions for clients to use the equipment appropriately. Participants who tried remote monitoring identified that clients often lacked motivation or had an abnormal mental state that hindered their ability to reliably use monitoring equipment:

That was interesting because [medical] community teams, where say people came in [to get treatment] then go back and then phone them (for physical observations). So do your obs [physical observations] and that by phone, whereas you can’t really rely on our client base to do that.

Participant 5

Participants had polarized opinions on the nature of the therapeutic relationship using audiovisual services. Some participants found it difficult to engage clients in therapeutic interventions and felt disconnected from them. On reflection, these participants identified that they had limited prior experience with audiovisual services or faced difficulty in accessing training programs. By contrast, participants who described good therapeutic relationships identified that training sessions, previous experience with audiovisual services, and existing therapeutic relationships with their client facilitated therapeutic engagement using audiovisual services:

There are some people who I've nursed on the ward, some people that I know from the community, so we always use those relationships. Imagine if I was sick and somebody says that [clinician] can call in to see you, I would be quite happy.

Participant 23

Participants were concerned that some clients with personality disorder diagnoses (eg, borderline, antisocial, histrionic, or narcissistic personality disorders) could engage in challenging behaviors. Clinicians found that these behaviors were difficult to identify and address using telehealth, as they were less likely to be able to identify transference and countertransference reactions and observe facial expressions and tones of voice. Furthermore, participants found that in-person contact created a safe therapeutic space for managing these behaviors, which clinicians found challenging to create by using audiovisual services:

Severe personality disorder in the forensic field, persons who can manipulate you, I wouldn’t trust on a virtual level. Again, there’s something, this gut feeling, I would trust myself more seeing a person face-to-face, but they can also manipulate that.

Participant 19

Participants identified that Māori clients, who preferred their native language (Te Reo Māori), faced additional barriers to engaging in audiovisual services. The participants recognized that the use of a client’s native language could facilitate understanding, connection, and therapeutic relationships. Clinicians found it difficult to access translators during lockdown, and when available, found it challenging to work with a translator using audiovisual technology. *Kaimahi* found that Māori clients preferred a combination of Te Reo Māori and English and subjectively had improved engagement with clinicians who spoke Te Reo Māori. Non-Māori clinicians reported improved engagement with Māori clients during audiovisual appointments when using Te Reo Māori phrases and *karakia* (blessings). Clinicians felt that this projected a sense of respect and cultural safety when in-person connections were not possible:

The use of language from a cultural perspective would be inviting and warm and would be a real opportunity to say I feel okay with this because I’ve engaged using a Māori kupu [greeting]. Something as basic as that which may just open a door a little bit. It’s about respect, integrity.

Participant 11

Some participants identified that clients with disabilities, such as vision and hearing impairments, would face difficulties with audiovisual service provision. They explained that it could be difficult to lip-read or use sign language effectively because of limitations in the video quality and resolution of facial features. In addition, audio quality could limit the client’s ability to follow discussions and, therefore, participate effectively. These groups would usually have a support person or translator present during in-person appointments, which clinicians found difficult to access during lockdowns on an audiovisual platform:

For one thing, people with hearing problems, particularly older people, they often do a lot of lipreading, and they look at the non-verbal cues and stuff quite a lot, so we didn’t have that.

Participant 12

## Discussion

### Principal Findings

The COVID-19 pandemic created challenges for the provision of mental health services. In the study regions, there was a rapid uptake of telehealth for remote service provision; however, in contrast to international literature [31,54], there was a sudden retreat from the telehealth service provision after the pandemic. Notably, the use of audiovisual methods for telehealth delivery was consistently low in our study regions. This study explored the views of 33 clinicians from 21 outpatient teams and integrated these findings with appointment data to develop an understanding of the factors contributing to telehealth uptake and retreat during the COVID-19 pandemic. This study focused on 6 key themes that contributed to the clinician’s decision to use telehealth services.

### Comparison With Prior Work

#### Overview

This study highlights the impromptu nature of telehealth service adoption by New Zealand public mental health services during lockdowns, which reverted to prelockdown practices. The PERCS Framework for Planning and Evaluating Remote Consultation Services provides a structure for understanding the adoption of telehealth without significant organizational planning [55]. This study identified a fluctuating tension for change, issues with preparedness, the need for training (skill development), risks and lack of resources (all aspects of digital maturity), and the recognition of digital inclusion (including clinicians).

#### Digital Maturity

Publicly funded mental health services in New Zealand face workforce issues, where clients have difficulty accessing physicians and clinical psychologists [41,56]. Participants in this study proposed that audiovisual service provision could be used to improve access to psychiatrists for clinical reviews and to provide timely advice on treatment from a distance. This is in keeping with international literature on the use of telehealth services to improve mental health care access to remote locations, where there is a lack of mental health services [57-60]. Furthermore, this study shows that the rate of missed appointments due to nonattendance decreased during the lockdown and with telehealth service provision. Audiovisual services can allow appointments to be conducted at short notice when clients or clinicians cannot attend in-person services. Timely access to clinicians and reduced time wasted by nonattendance can improve service efficiency [61-63]. Audiovisual services have the potential to complement current service provision and improve service efficiency, which could improve access to clinicians and reduce the demand on the current mental health workforce.

Audiovisual service provision has not been successfully adapted to provide physical monitoring services. Participants identified a lack of support and equipment to loan to clients to monitor vital signs (eg, blood pressure monitors). Physicians have used audiovisual services in conjunction with equipment loaned to clients to support physical monitoring from a distance during lockdowns [64,65]. Investing in medical equipment to loan to clients could improve nurses’ monitoring capabilities and reduce the need for clients and clinicians to travel. This could broaden the scope of audiovisual service delivery and change the model of service provision to allow clients to remain in the comfort of their homes.

This study highlights the resource limitations experienced by public mental health services in New Zealand, which contributed to the digital exclusion for individual clinicians, clients, and teams. Participants described a lack of basic hardware, software, private spaces, and technical support to effectively use audiovisual services. *Kaimahi* described more severe resource constraints for Māori clinicians and clients, limiting their access to audiovisual and telephone services and manifesting as fewer telehealth appointments with Māori populations. This is in line with disparities in Indigenous mental health care access [33,66]. Furthermore, national figures on digital exclusion [67,68] highlight the disparate access to digital technology for the Māori and Pacific populations. There is a need to address resource limitations for both clinicians and clients to implement telehealth services and to reduce disparities in accessing mental health care faced by Indigenous populations.

#### Digital Inclusion

Clinicians lacked confidence in adopting audiovisual service provision because of the difficulties in conducting consultations. Clinicians found it challenging to interpret subtle visual cues, conduct physical examinations, and develop rapport with their clients. These barriers contradict international research on telehealth efficacy in mental health settings [12-14], which often includes participants with specialist training and experience in audiovisual service provision. One possible explanation for the predominantly negative views expressed in this study could be inadequate training on the use of audiovisual services. In the study regions, clinicians who were familiar with using telehealth or who received training did not experience the same level of difficulty in conducting consultations. Therefore, it is important to establish local training programs for mental health clinicians to develop appropriate skills to use audiovisual services effectively and support clinicians with funding and telehealth training time. However, we do acknowledge the importance of understanding intentional nonengagement with technology by clinicians and the importance of engaging in human-to-human contact as part of maintaining an effective therapeutic relationship (particularly given the risks that therapeutic alliances may be impaired when services are delivered online) [69,70].

This study identified that clients with severe illness, or who posed a risk to themselves or others, were not appropriate to review by telehealth services. There is a lack of clinical trials examining telehealth use for clients with severe mental illness, schizophrenia, borderline personality disorder, and clients experiencing a mental health crisis (emergency department). This could reflect the concerns around the capacity to provide consent in these groups, which would limit their inclusion in research [71,72]. Participants navigated through these difficulties by providing a hybrid approach to service delivery, with clinicians attending in person to support decision-making and manage client risk. Further research is required on the suitability of telehealth service provision and hybrid service approaches for higher-risk populations. This could support clinician decision-making regarding when telehealth services are appropriate to use and the limitations of telehealth service provision. It could also help inform discussions on how to engage clients in services and inform considerations related to improving client choice in care. Such research is particularly important, given the wide variety of research highlighting the potential for telehealth to enable clients to have a choice in their care [73,74].

### Implications for Policy and Practice

Textbox 3 provides key recommendations for clinicians and health system management. For clinicians, these recommendations relate to improving their knowledge about how to operate and deliver telehealth services to improve engagement with clients. For health system managers, these recommendations primarily relate to funding and maintaining telehealth-capable systems, clinicians, and wider resourcing.

In addition, our study highlights the need for continued engagement with Indigenous Māori populations around the value of telehealth, particularly given the later presentation of Māori populations to mental health services and differential engagement with telehealth [66,75,76]. Such findings align with the global research on Indigenous populations and furthers discussion on the need for supporting Indigenous-led delivery of mental health services [33,77,78], this time in the telehealth arena.

**Key recommendations for clinicians and health system management regarding telehealth service.**

**Clinicians**

- Review available systems and telehealth resources to determine if they meet current standards for conducting telehealth consultation.
- Undertake training on the effective use of telehealth for mental health service provision.
- Access training on establishing a telehealth clinic and appointments.
- Engage clients in conversation about their service access, including barriers.
- Inform clients about telehealth to allow clients to choose their preferred mode of service provision.

**Health system managers**

- Fund and maintain telehealth-capable systems.
- Engage with outpatient clinicians to improve access to resources, hardware, and new technology to facilitate telehealth service provision.
- Provide funding and leave allocation to attend training on telehealth service provision.
- Create a local training program for clinicians to learn how to use telehealth effectively.
- Engage with Indigenous clients and the clinicians who provide Indigenous services to them to discuss how telehealth services can be adapted for Indigenous people needs to improve access to telehealth service.
- Develop information technology services and improve resources to provide technical support for clinicians.

### Limitations

This study generates valuable recommendations for the implementation of regional telehealth-based mental health services and is applicable to business as usual and pandemic settings. A strength of this study is its use of population-level data, meaning that we have an accurate picture of activities occurring in our study region rather than a sample requiring statistical inference of probability [79]. The ability to extrapolate the findings to other regions may be limited. The data set used in this study was only the client activity data record for the study region. However, the data set did not provide information on the purpose or content of appointments. Therefore, it was not possible to identify whether specific changes in clinicians’ consultation approaches occurred; rather, we could only infer that the changes occurred based on qualitative findings, supported by changes in appointment duration and frequency.

Similarly, the data set did not contain clinician demographic information to be included in this study. There was no feasible method for obtaining deidentified clinician demographic information to then match unique identifiers with the activity data. This limited the triangulation of qualitative themes regarding the effect of clinician age and ethnicity on telehealth use.

Notably, although changes occurred in the study regions’ data collection practices regarding gender and ethnicity during this project, there remains little quantitative data on ethnicity and refugee status. Clinicians also did not specifically speak about these populations, potentially indicating a lack of recognition of their specific telehealth or mental health needs. This engenders debate about the need for culturally specific services for other minority group cultures in the study region, such as Asian or Middle Eastern people, who collectively account for >17% of the New Zealand population [80].

### Future Research

In many parts of the world, telehealth has provided solutions to health care access. However, there remain circumstances where its use may fail to meet population needs, including in people with disabilities [81]. Therefore, further evaluation of its use is necessary after the pandemic, with an eye to understanding how telehealth use has evolved culturally with a growing acceptance of people working and delivering care from a distance. Future research should include evaluating the reasons why specific clinicians have continued to incorporate telehealth into their usual practice. Alongside this, further research is needed on the utility of telehealth for clients presenting with severe mental illness; risks (associated with their illness); and disabilities (hearing, visual, and intellectual) and clients from other cultural minority groups. Having this understanding is particularly important to inform any decision-making regarding telehealth’s continued utility. We recommend that such research is conducted alongside these populations and is informed by data that accurately capture important demographics and these clients’ voices.

### Conclusions

Telehealth was used during the pandemic, driven by the necessity to provide mental health services from a distance. Following the lockdown, telehealth fell out of favor in the study region, despite significant potential benefits for usual practice, including improved access to health care and client choice and reduced service inefficiency. Mental health services in the study region are moving back to in-person service provision, perhaps due to factors such as a lack of training, support, and resourcing, as suggested by our research participants. For telehealth to be successfully implemented, there is a need to invest in clinicians and mental health teams to develop effective telehealth-capable services.

## Appendix

Multimedia Appendix 1 Demographic questionnaire.

## Data Availability

The data sets analyzed during this study are the properties of the relevant mental health services and are not publicly available to protect human privacy. The Memorandum of Understanding governing the use of these data does not permit public sharing of the data set.
