# Correction: Global Implications From the Rise and Recession of Telehealth in Aotearoa New Zealand Mental Health Services During the COVID-19 Pandemic: Mixed Methods Study

**DOI:** 10.2196/64385

**Published:** 2024-07-30

**Authors:** Benjamin Werkmeister, Anne M Haase, Theresa Fleming, Tara N Officer

**Affiliations:** 1 School of Health, Te Herenga Waka Victoria University of Wellington Wellington New Zealand; 2 Department of Psychological Medicine University of Otago (Wellington) Wellington New Zealand; 3 Te Whatu Ora Wellington New Zealand; 4 School of Nursing, Midwifery, and Health Practice, Te Herenga Waka Victoria University of Wellington Wellington New Zealand

In “Global Implications From the Rise and Recession of Telehealth in Aotearoa New Zealand Mental Health Services During the COVID-19 Pandemic: Mixed Methods Study” ([JMIR Form Res 2023;7: e50486]) the authors noted one error:

1. In the Abstract, Methods, the following sentence:

In total, 4,117,035 observations were analyzed between October 1, 2019, and August 1, 2022.

Has been revised to:

In total, 4,117,035 observations were analyzed between September 2, 2019, and August 1, 2022.

2. In Methods, Activity data – Overview, the following sentence:

This data set represented population-level data and included all clinical activities (4,117,035 observations) undertaken by outpatient mental health clinicians in the study regions between October 2019 and August 2022 to allow comparison of telehealth use between prelockdown, lockdown, and postlockdown periods.

Has been revised to:

This data set represented population-level data and included all clinical activities (4,117,035 observations) undertaken by outpatient mental health clinicians in the study regions between September 2019 and August 2022 to allow comparison of telehealth use between prelockdown, lockdown, and postlockdown periods.

3. Also in Methods, Table 1 – column 2, row 2, the following sentence:

October 1, 2019 (12 AM)

Has been revised to:

September 2, 2019 (12 AM)

The correction will appear in the online version of the paper on the JMIR Publications website on July 30, 2024, together with the publication of this correction notice. Because this was made after submission to PubMed, PubMed Central, and other full-text repositories, the corrected article has also been resubmitted to those repositories.

