# Supplementary material for: Global Implications From the Rise and Recession of Telehealth in Aotearoa New Zealand Mental Health Services During the COVID-19 Pandemic: Mixed Methods Study
Source: JMIR Form Res. 2023 Sep 22;7:e50486. doi: 10.2196/50486 (PMC10519279; doi:10.2196/50486)
Supplement: Multimedia Appendix 1 [file formative_v7i1e50486_app1.docx]

## **Telehealth, protect the bubble: mental health service delivery during COVID-19**

## Demographic questions for participants

1. What is your gender?

- Male
- Female
- Gender diverse

1. What age group do you belong to?

- Less than 25 years old
- 25-34 years old
- 35-44 years old
- 45-54 years old
- 55-64 years old
- 65 years old or over

1. What ethnic group or groups do you belong to? (Please tick all that apply to you)

- New Zealand European
- Māori
  - Iwi/ hapū:
- Samoan
- Cook Island Maori
- Tongan
- Niuean
- Chinese
- Indian
- Other such as Dutch, Japanese, Tokelauan. Please state: ______________

1. I consider myself very familiar with using Zoom or other video conferencing apps:

- Strongly disagree
- Disagree
- Neither agree nor disagree
- Agree
- Strongly agree

For clients:

1. How long have you received services from MHAIDS?

- 0-6 months
- 6 months – 1 year
- Longer than 1 year
- On and off for many years using the one service
- On and off for many years using multiple different MHAIDS services

1. I have access to: (Please tick all that apply to you)

- Telephone
- Internet

For clinicians:

1. How many years have you been practising in mental health? _____
2. How many years have you been working in your current team? ______
